# Functional Outcomes of Minimally Invasive Titanium Elastic Nailing for Displaced Midshaft Clavicle Fractures: A Prospective Study

**DOI:** 10.7759/cureus.111888

**Published:** 2026-07-01

**Authors:** Jayant Kothale, Vishal Kumar, Mohamed P Azlam, Ravi Sharma

**Affiliations:** 1 Orthopedics, Government Medical College, Seoni, IND; 2 Orthopedics, Netaji Subhas Medical College and Hospital, Patna, IND; 3 Orthopedics, Netaji Subhash Chandra Bose (NSCB) Medical College and Hospital, Jabalpur, IND

**Keywords:** functional outcome assessment, intramedullary interlock nailing, midshaft clavicle fracture, titanium elastic nail, titanium elastic nailing system (tens)

## Abstract

Background: Displaced midshaft clavicle fractures account for the majority of all clavicular injuries. Titanium elastic nailing system (TENS) offers a minimally invasive alternative to plate fixation. The primary objective was to assess the efficacy and safety of minimally invasive TENS by evaluating functional, clinical, and radiological outcomes, including union and complication rates, in displaced midshaft clavicle fractures.

Methods: This prospective study included 30 patients (25 male and five female patients; mean age 38.06 years) with displaced midshaft clavicle fractures treated with TENS between December 2019 and August 2021. Fractures were classified using Arbeitsgemeinschaft für Osteosynthesefragen/Orthopedic Trauma Association classification as 18 Type 15-B1 and 12 Type 15-B2. Follow-up occurred at one, three, and six months. Outcomes were assessed using the Constant-Murley score, Disabilities of the Arm, Shoulder and Hand (DASH) score, and Visual Analog Scale (VAS).

Results: Closed reduction succeeded in 25 (83.3%) patients. Mean surgical time was 31.67 minutes. Clinical union occurred at 6.7 weeks and radiological union at 8.43 weeks. At six months, the mean Constant score was 91.00 ± 4.51, with 26 (86.7%) patients having excellent results; the mean DASH score was 12.30 ± 2.93, and the mean VAS score was 3.53 ± 1.17. Hardware removal was required in nine (30.0%) patients, supraclavicular paresthesia occurred in five (16.7%) patients, and minimal shortening occurred in seven (23.3%) patients. One patient developed a nonunion. No deep infections or hardware breakage developed.

Conclusions: Minimally invasive titanium elastic nailing appears to be a useful treatment option for displaced midshaft clavicle fractures, with favorable short-term functional and radiological outcomes in this prospective single-arm study. The procedure was associated with timely union and acceptable complication rates, although hardware-related problems such as nail migration and implant removal were not uncommon.

## Introduction

Clavicular fractures represent 2%-10% of all adult fractures, with 80% occurring in the midshaft region [1]. Nonoperative treatment is associated with 15%-18% nonunion rates in displaced fractures, along with malunion, shortening exceeding 20 mm, persistent pain, and shoulder dysfunction [2]. Clavicular shortening of 1-2 cm significantly impairs shoulder biomechanics, reducing strength and limiting overhead activities [3].

Open reduction and internal fixation with plates has historically been widely used and regarded as a standard operative treatment for displaced midshaft clavicle fractures; however, contemporary randomized trials and meta‑analysis indicate that both plate fixation and intramedullary fixation achieve high union rates and similar long‑term functional outcomes, with intramedullary techniques often showing shorter operative time, smaller incisions, and fewer wound complications [4]. Complication rates with plating range from 15% to 25%, with hardware removal required in up to 40% [5]. Intramedullary fixation using titanium elastic nailing system (TENS) has emerged as a minimally invasive alternative. The clavicle's S-shaped curvature allows elastic nails to achieve three-point fixation, providing load-sharing stability that promotes callus formation through controlled micromotion [6]. TENS offers smaller incisions, minimal dissection, preserved fracture hematoma, reduced neurovascular risk, lower infection rates, and superior cosmesis [7]. However, TENS may be associated with implant‑specific complications, including telescoping and migration, hardware irritation, and reduced rotational stability in comminuted fractures, which may necessitate secondary procedures and careful attention to implant diameter, end‑cap use, and nail positioning [8].

Comparative studies demonstrate favorable TENS outcomes compared with plating, including a faster return to activities, comparable union rates, and fewer complications [9]. However, concerns exist regarding efficacy in comminuted fractures due to potential telescoping and nail migration [10]. Despite growing evidence supporting TENS, comprehensive prospective data from Indian populations remain limited. The primary objective of this study was to assess the efficacy and safety of minimally invasive TENS by evaluating functional, clinical, and radiological outcomes, including union and complication rates, in displaced midshaft clavicle fractures. The secondary objective was to explore whether outcomes differed between simple and comminuted fracture patterns.

## Materials and methods

Study design and setting

This prospective study was conducted in the Department of Orthopedics, Netaji Subhash Chandra Bose Medical College and Hospital, Jabalpur, India, from December 2019 to August 2021. This was a prospective single‑arm series without a control group, enrolling all eligible patients treated during the study period; no formal a priori sample size calculation was performed. The study protocol was approved by the Institutional Ethics Committee, Netaji Subhash Chandra Bose Medical College and Hospital, Jabalpur (approval letter no. IEC/NSCBMC/19/22), and written informed consent was obtained from all participants prior to enrollment.

Patient selection

Patients aged 22 years and above with completely displaced midshaft clavicle fractures (Arbeitsgemeinschaft für Osteosynthesefragen (AO)/Orthopedic Trauma Association (OTA) Type 15-B) without cortical contact between fragments were included. Acute fractures (less than three weeks old) without neurovascular injury, no medical contraindications to anesthesia, and a willingness to comply with follow-up were required. Exclusion criteria included fractures involving medial or lateral thirds, pathological fractures, open fractures, neurovascular injury, Glasgow Coma Scale ≤12, ipsilateral upper extremity fractures, prior shoulder surgery, preexisting shoulder dysfunction, contralateral clavicle fracture, and inability to comply with rehabilitation.

We included patients aged ≥22 years to ensure skeletal maturity of the clavicle, as complete fusion of the medial clavicular epiphysis generally occurs around 22 years of age or later.

Preoperative evaluation

All patients underwent comprehensive clinical assessment including detailed injury history, physical examination, and neurovascular status documentation. Standard anteroposterior and 45° cephalic tilt radiographs of the affected clavicle were obtained for fracture classification. Laboratory investigations included complete blood count, renal function tests, coagulation profile, viral serology (HIV, hepatitis B and C), chest radiography, and electrocardiography. Fractures were classified according to the AO/OTA classification system: Type 15-B1 (simple transverse/oblique) and Type 15-B2 (wedge/comminuted fractures).

Surgical technique

All procedures were performed under general anesthesia or interscalene block. Patients were positioned in beach chair or supine position on a radiolucent operating table with a bolster placed interscapularly to provide shoulder extension and enhance clavicular prominence. The operative field was prepared from the midline chest to the base of the neck, including the entire upper extremity to allow intraoperative arm manipulation. Image intensification was used throughout the procedure.

A 1-2 cm longitudinal skin incision was made just medial to the sternoclavicular joint. After dissecting the platysma and underlying fascia, the medullary canal was opened using a bone awl approximately 1-2 cm lateral to the sternoclavicular joint, angled 30° to the coronal plane under fluoroscopic guidance. Care was taken to avoid dorsal cortex perforation. An appropriately sized titanium elastic nail (2.5 mm diameter) was selected based on contralateral clavicle measurement and advanced through the medullary canal with oscillating movements until reaching the fracture site.

Fracture reduction was attempted using direct manual pressure on fragments combined with arm manipulation. When closed reduction failed, percutaneous pointed reduction clamps were applied, or a small incision (2-3 cm) was made over the fracture site for direct manipulation. Following satisfactory reduction confirmed by fluoroscopy, the nail was advanced across the fracture to within 2 cm of the acromioclavicular joint, avoiding dorsolateral cortex perforation. In selected cases (n = 6), TENS with end-cap fixation was utilized for enhanced medial stability. An end‑cap was used in patients with comminuted AO/OTA 15‑B2 fractures and/or a relatively wide medullary canal, or when intraoperative assessment indicated a tendency for medial nail migration. The medial nail end was cut flush with the bone, leaving sufficient length for future removal. Wounds were irrigated, closed in layers, and sterile dressings applied.

Postoperative protocol and rehabilitation

Postoperatively, patients received intravenous antibiotics for three days, analgesics, and proton pump inhibitors. An arm-pouch sling was provided for two weeks, with wound inspection on day 2 and suture removal on day 11. Rehabilitation progressed through phases: weeks 0-2 involved assisted passive mobilization four times daily with continuous sling use; weeks 2-6 featured active shoulder mobilization after sling discontinuation; week 6 onward permitted progressive activities as tolerated; and three months onward allowed resumption of heavy lifting and strenuous activities.

Follow-up and outcome assessment

Outcome measures included the Constant-Murley Score (0-100 points) [11], which assesses pain, activities of daily living, range of motion, and strength; the Disabilities of the Arm, Shoulder and Hand (DASH) Score (0-100) [12], which evaluates upper extremity function with higher scores indicating greater disability; and the Visual Analog Scale (VAS, 0-100 mm) [13] for pain assessment.

Patients were evaluated at one, three, and six months postoperatively with clinical examination assessing pain, range of motion, fracture-site tenderness, and functional recovery. Radiographic evaluation confirmed union, implant position, and alignment. Hardware removal was performed after complete radiological union in symptomatic patients. Radiological union was defined as bridging callus across three cortices with fracture line obliteration, and clinical union was defined as absence of tenderness with pain-free movement. Complications included hardware prominence, infection, nonunion, nerve injury, and migration.

Statistical analysis

Data were analyzed using descriptive statistics (mean, standard deviation, percentages). Repeated‑measures analysis of variance was used to assess changes in Constant, DASH, and VAS scores across follow‑up; independent‑samples t‑tests were used to compare continuous variables between fracture types; Fisher’s exact test or two‑sample Z‑tests were used to compare categorical outcomes. Statistical significance was set at p < 0.05. Analysis was performed using Statistical Package for the Social Sciences Version 25 (IBM Corp., Armonk, NY) statistical software.

## Results

Patient demographics and fracture characteristics

Thirty patients completed six-month follow-up (25 male and five female patients; mean age 38.06 years, range 22-60 years). Right-sided fractures occurred in 16 (53.3%) patients and left-sided fractures in 14 (46.7%) patients. Road traffic accidents accounted for 22 (73.3%) injuries, followed by falls from stairs in five (16.7%), falls from height in two (6.7%), and falls on the ground in one (3.3%) patient.

Surgical details

According to AO/OTA classification, 18 (60.0%) patients had Type 15-B1 simple fractures, while 12 (40.0%) had Type 15-B2 comminuted fractures. All patients received 2.5 mm titanium elastic nails, including 24 standard TENS and six TENS with end-cap. Closed reduction succeeded in 25 (83.3%) patients, including 16/18 (88.9%) in Type 15-B1 and 9/12 (75.0%) in Type 15-B2 fractures (p = 0.364). Mean surgical time was 31.67 minutes, including 28 minutes for Closed Reduction Internal Fixation and 42 minutes for Open Reduction and Internal Fixation (Table 1).

**Table 1:** Demographic and surgical characteristics of study subjects (n = 30) AO: Arbeitsgemeinschaft für Osteosynthesefragen; ORIF: open reduction internal fixation; CRIF: closed reduction internal fixation

Variable	Value, n (%)
Gender	
Male	25 (83.3%)
Female	5 (16.7%)
Fracture side	
Right	16 (53.3%)
Left	14 (46.7%)
AO classification	
Type 15-B1 (simple)	18 (60.0%)
Type 15-B2 (comminuted)	12 (40.0%)
Reduction method	
Closed/mini-open (CRIF)	25 (83.3%)
Open (ORIF)	5 (16.7%)

Union and radiological outcomes

Clinical union occurred at a mean of 6.7 weeks (range 6-9 weeks), and radiological union at 8.43 weeks (range 7-11 weeks). Type 15-B2 fractures demonstrated earlier radiological union (8.00 weeks) than Type 15-B1 fractures (8.50 weeks; p = 0.037). Postoperative shortening of 2-4 mm occurred in seven (23.3%) patients and was significantly higher in comminuted fractures, 6/12 (50.0%), than in simple fractures, 1/18 (5.5%), though without functional impact (Table 2). Although Type 15-B2 fractures showed a slightly earlier mean radiological union than Type 15-B1 fractures, the absolute difference was only 0.5 weeks and is unlikely to be clinically important. Given the small sample size and exploratory subgroup analysis, this finding should be interpreted cautiously and may reflect sampling variability rather than a true difference in healing biology.

**Table 2:** Comparison of clinical union and radiological union among study subjects ^*^Statistically significant (p < 0.05) SD: standard deviation; NS: not significant

Fracture type	Clinical union (mean ± SD), weeks	Radiological union (mean ± SD), weeks	Test	t value	p value	Result
Type 15-B1 (n = 18)	6.89 ± 0.76	8.50 ± 0.62	Independent t-test	t = 1.75 (clinical)	0.09	NS
Type 15-B2 (n = 12)	6.41 ± 0.67	8.00 ± 0.60	Independent t-test	t = 2.20 (radiological)	0.037	Significant^*^

Functional outcomes

At six months, the mean Constant score was 91.00 ± 4.51, with 26 (86.7%) patients having excellent results (86-100) and four (13.3%) having good results (71-85). The mean DASH score was 12.30 ± 2.93, and the mean VAS score was 3.53 ± 1.17. Functional outcomes showed no significant differences between fracture types: The Constant scores for Type 15-B1 were 90.44 ± 3.97, while Type 15-B2 had scores of 91.83 ± 5.29 (p = 0.418). The DASH scores were 12.39 ± 2.75 for Type 15-B1 and 12.17 ± 3.27 for Type 15-B2 (p = 0.842). The VAS scores were 3.67 ± 1.14 for Type 15-B1 and 3.33 ± 1.23 for Type 15-B2 (p = 0.453). Patients returned to daily activities at 3.38 weeks and professional activities at 6.93 weeks (Table 3).

**Table 3:** Functional outcome measures at follow-up ^a^Constant score was assessed at one, three, and six months Values are presented as mean ± standard deviation. Repeated-measures ANOVA was used to assess changes in Constant-Murley, DASH, and VAS scores over time; the corresponding F-statistic is reported. Statistical significance was set at p < 0.05 RM-ANOVA: repeated measures analysis of variance; DASH: Disabilities of Arm, Shoulder and Hand; VAS: Visual Analog Scale

Outcome measure	Follow-up values			Statistical test	Test statistic	p value
	1 month	3 months	6 months			
Constant scoreᵃ	71.77 ± 4.32	83.13 ± 3.85	91.00 ± 4.51	RM-ANOVA	F(2,57) = 0.000	<0.001
DASH score	21.17 ± 5.48	11.33 ± 4.12	12.30 ± 2.93	RM-ANOVA	F(2,57) = 0.000	<0.001
VAS score	6.90 ± 0.80	8.27 ± 0.78	3.53 ± 1.17	RM-ANOVA	F(2,57) = 0.000	<0.001

Complications

Hardware prominence requiring removal occurred in nine (30.0%) patients at a mean of 101 days. Supraclavicular paresthesia developed in five (16.7%) patients, all in open reduction cases, with partial improvement following neuropathic therapy. Nail migration occurred in nine (30.0%) patients and was significantly higher in comminuted fractures, 7/12 (58.3%), than in simple fractures, 2/18 (11.1%) (p = 0.005). Minimal shortening of 2-4 mm occurred in seven (23.3%) patients, predominantly in comminuted fractures, 6/12 (50.0%), compared with simple fractures, 1/18 (5.5%) (p = 0.004). One patient, 1 (3.3%), developed nonunion and required revision surgery. One patient, 1 (3.3%), developed superficial infection. No deep infections, hardware breakage, or refractures occurred (Table 4).

**Table 4:** Comparison of complications among both fracture types among study subjects ^*^Statistically significant (p < 0.05). Z-test used for two-proportion comparison (shortening and nail migration); Fisher's exact test used for all other comparisons owing to small cell frequencies

Complication	Type 15-B1 (n = 18), n (%)	Type 15-B2 (n = 12), n (%)	Total, n (%)	Test used	Test statistic	p value
Hardware removal	4 (22.2%)	5 (41.7%)	9 (30.0%)	Fisher's exact	Exact = 0.622^*^	0.269
Nail migration	2 (11.1%)	7 (58.3%)	9 (30.0%)	Z-test (two-proportion)	Z = 2.76	0.005^*^
Supraclavicular paresthesia	3 (16.7%)	2 (16.7%)	5 (16.7%)	Fisher's exact	Exact = 1.000	1.000
Shortening (2-4 mm)	1 (5.5%)	6 (50.0%)	7 (23.3%)	Z-test (two-proportion)	Z = 2.819	0.004^*^
Nonunion	1 (5.5%)	0 (0.0%)	1 (3.3%)	Fisher's exact	Exact = 1.000	1.000
Superficial infection	0 (0.0%)	1 (8.3%)	1 (3.3%)	Fisher's exact	Exact = 0.400	0.400
Deep infection	0 (0.0%)	0 (0.0%)	0 (0.0%)	-	-	-
Hardware breakage	0 (0.0%)	0 (0.0%)	0 (0.0%)	-	-	-
Refracture	0 (0.0%)	0 (0.0%)	0 (0.0%)	-	-	-

## Discussion

This prospective study demonstrates that minimally invasive titanium elastic nailing is highly effective for displaced midshaft clavicle fractures, yielding excellent functional outcomes, rapid union, and acceptable complications. Our findings align with contemporary literature supporting TENS as a viable alternative to plate fixation [14-16].

The demographic profile reflects typical epidemiology, with male predominance (83.3%) and a mean age of 38 years, consistent with the literature [17]. Road traffic accidents constituted 73.3%, mirroring high-energy trauma mechanisms in developing countries [18]. The 83.3% closed reduction success compares favorably with recent studies reporting 60%-70% rates [19], reflecting surgeon experience and early intervention. Mean surgical time (31.67 minutes) was substantially shorter than plating (60-90 minutes), corroborating TENS operative efficiency [20].

Our mean radiological union (8.43 weeks) compares favorably with recent studies reporting 10-12 weeks [21], suggesting TENS-associated micromotion enhances healing. The earlier union in comminuted fractures (8.00 vs. 8.50 weeks, p = 0.037) contradicts assumptions that comminution delays healing, likely reflecting abundant callus formation with flexible fixation [20,22]. The 96.7% union rate aligns with systematic reviews reporting 97%-98% union with intramedullary nailing [23].

The mean Constant score (91.00 ± 4.51), with 86.7% excellent results, demonstrates superior functional restoration, comparable to contemporary series reporting scores ranging from 81 to 94 [24]. DASH score (12.30 ± 2.93) indicates minimal disability. Importantly, no significant functional differences existed between simple and comminuted fractures (p>0.05), challenging the contraindications to TENS in comminuted patterns. Rapid return to activities (3.38 weeks daily, 6.93 weeks professional) represents major advantages over nonoperative treatment (8-12 weeks) and plating (8-16 weeks) [6].

Hardware prominence requiring removal (30%) primarily affected comminuted fractures due to telescoping (nail migration 58.3% in Type 15-B2 vs. 11.1% in Type 15-B1, p = 0.005). Strategies include TENS with an end cap for comminuted fractures and flush medial nail cutting. Supraclavicular paresthesia (16.7%), exclusively in open reductions, underscores the importance of meticulous technique. Minimal shortening (2-4 mm, 23.3%) had no functional impact, consistent with biomechanical studies showing shortening <15 mm does not compromise kinematics [7]. The absence of deep infections (0%), hardware breakage (0%), and refractures (0%) compares favorably with plate fixation rates of 5%-8% for infection and 2%-4% for refracture [25].

The present study has several strengths, including its prospective design, clearly defined eligibility criteria, standardized operative and rehabilitation protocols, and use of validated outcome measures such as the Constant-Murley score, DASH score, and VAS. The inclusion of both simple and comminuted fracture patterns also provides clinically useful information regarding the applicability of TENS across different fracture morphologies. However, the study has important limitations. These include the small sample size, single-center design, absence of a comparative group, and a relatively short six-month follow-up, which may not capture late complications or long-term functional outcomes. In addition, no preoperative functional scores were obtained, limiting the ability to quantify the magnitude of postoperative improvement, and the lack of formal sample size calculation means that subgroup comparisons should be interpreted as exploratory. The relatively high rates of nail migration and hardware removal also warrant cautious interpretation, as these complications may influence the overall assessment of treatment success. Because this was a single-arm observational study, conclusions regarding superiority over plate fixation or nonoperative treatment cannot be drawn. Therefore, TENS should be considered a useful minimally invasive treatment option in appropriately selected patients rather than a universally preferred first-line treatment. Future multicenter randomized studies with larger sample sizes and longer follow-up are needed to confirm these findings and define the optimal role of TENS in displaced midshaft clavicle fractures.

## Conclusions

Minimally invasive titanium elastic nailing appears to be a useful treatment option for displaced midshaft clavicle fractures, with favorable short-term functional and radiological outcomes in this prospective single-arm study. The procedure was associated with timely union and acceptable complication rates, although hardware-related problems such as nail migration and implant removal were not uncommon. While the findings support the feasibility of TENS in appropriately selected patients, the absence of a comparative group, the small sample size, and the short follow-up period prevent any firm conclusion regarding superiority over plate fixation or nonoperative treatment. Larger multicenter randomized studies with longer follow-up are needed to better define the role of TENS across different fracture patterns.
